# Supplementary material for: Dynamic academic networking concept and its links with English language skills and research productivity–non-Anglophone context
Source: PLoS One. 2021 Feb 2;16(2):e0245980. doi: 10.1371/journal.pone.0245980 (PMC7853504; doi:10.1371/journal.pone.0245980)
Supplement: S1 Appendix — (DOCX) [file pone.0245980.s001.docx]

# S1 APPENDIX

**The survey questions**

| **Construct** | **Items type, code and wording** | **Items source and reliability** |
| --- | --- | --- |
| **Dynamic networking** | **Likert scale: 1 – definitely disagree; 7 - definitely agree**  **3_9_Networking_5** - In recent years the number of researchers I socialize with has increased significantly  **3_10_Networking_1** - In recent years there was a significant increase of number of scholars I cooperate with in research and publishing  **3_11_Networking_2 -** I try systematically to broaden the area of my research contacts through initiating contacts with new people at national and international conferences  **3_12_Networking_3-** I usually search for new research partners (e.g. through social media, cooperation offers)  **3_13_Networking_4** - I systematically evaluate the usability of my current research contacts (e.g. taking into consideration such benefits as joint publications) | Adapted from [90]  Cronbach's Alpha = 0.860; AVE = 0.629 |
| **Home network** | **Likert scale: 1 – definitely disagree; 7 - definitely agree**  **3_1_Home_net_1-** I have a well-developed network of fellow researchers from my University who I talk with about research projects  **3_2_Home_net_2 -** I keep in touch with fellow researchers from my University, with whom I cooperate in research and publications | Adapted from [91]  Cronbach's Alpha = 0.861; AVE = 0.878 |
| **Corporate network** | **Likert scale: 1 – definitely disagree; 7 - definitely agree**  **3_7_Corpo_net1 -** I systematically cooperate with business practitioners (e.g. managers, entrepreneurs) in various research and expert projects  **3_8_Corpo_net2** - I systematically cooperate with business practitioners that has some impact on my research publications | Adapted from [91]  Cronbach's Alpha = 0.914; AVE = 0.918 |
| **English language skills** | **Likert scale: 1 – definitely disagree; 7 - definitely agree**  **2_10_English_oral -** In comparison to other researchers at my faculty, I have good English speaking skills  **2_11_English_write -** In comparison to other researchers at my faculty, I have good English writing skills | Developed for this study  Cronbach's Alpha = 0.960; AVE = 0.961 |
| **Productivity** | **Please specify your scientific outputs with regard to:**  **6_5_Journal_papers_2_years -** Number of publications in blind-reviewed journals within last two years …  **6_6_External_research_grants** - Number of external grants received for financing research projects …. | Adapted from [79]  Formative measure |
| **Teaching load** | **7_7_Teaching_load -** Please specify the typical number of teaching hours you provide as a university teacher per year  **…..** | Adapted from [87, 88] |
| **Resource support** | **Likert scale: 1 – definitely disagree; 7 - definitely agree**  **5_1_Resources_general** – I have an access to appropriate resources at my university  **5_3_Resources_office** – I have appropriate office to do research at my university | Adapted from [87, 88]  Cronbach's Alpha = 0.651; AVE = 0.735 |
| **Character** | **Likert scale: 1 – definitely disagree; 7 - definitely agree**  **1_1Personality –** I am a person that is always prepared | Simple scale inspired by [92, 93] |
| **Culture** | **Likert scale: 1 – definitely disagree; 7 - definitely agree**  **5_9_uni_productivity_pressure –** my university has strong demands with regard to research productivity | Simple scale developed for this study |
| **Employment time** | **Please specify:**  **7_8_Employment_at_current_uni –** years of employment at current university ….  **7_9_Employment_general –** years of employment in academia in general … | Developed for this study  Cronbach's Alpha = 0.887; AVE = 0.894 |
| **Academic rank** | **Please specify what is your current position at the university:**   1. PhD student/part time employment 2. Research assistant 3. Assistant professor 4. Assistant professor with PhD 5. Associate professor 6. Full professor | Simple scale developed for this study |
| **Age** | **Please specify your age: ….** | Developed for this study |

**The characteristics of research participants**

**Main universities included in the survey sample**

|  | number  of informants | Percent |
| --- | --- | --- |
| University of Economics in Katowice | 30 | 15,15 |
| University of Szczecin | 26 | 13,13 |
| University of Lodz | 16 | 8,08 |
| University of Economics in Poznań | 11 | 5,56 |
| Warsaw University of Life Sciences- SGGW | 11 | 5,56 |
| University of Gdansk | 10 | 5,05 |
| SGH Warsaw School of Economics | 9 | 4,55 |
| Wroclaw University of Economics | 9 | 4,55 |
| University of Warsaw | 9 | 4,55 |
| Kozminski University | 8 | 4,04 |
|  |  | 0,00 |
| University of Economics in Cracov | 8 | 4,04 |
| University of Zielona Góra | 8 | 4,04 |
| University of Warmia and Mazury | 6 | 3,03 |
| Częstochowa Univerity of Technology | 7 | 3,54 |
| Silesian University of Technology | 7 | 3,54 |
| Other universities | 23 | 11,62 |
| Total | 198 | 100,00 |

**Contract type**

|  | | Frequency | Percent |
| --- | --- | --- | --- |
| Valid | Indefinite | 130 | 65.7 |
|  | Definite | 68 | 34.3 |
|  | Total | 198 | 100.0 |

**Position at home faculty**

|  | | Frequency | Percent | Valid Percent | Cumulative Percent |
| --- | --- | --- | --- | --- | --- |
| Valid | PhD student/part time employment | 5 | 2.5 | 2.5 | 2.5 |
|  | Research assistant | 122 | 61.6 | 61.6 | 64.1 |
|  | Assistant professor | 42 | 21.2 | 21.2 | 85.4 |
|  | Assistant professor with PhD | 17 | 8.6 | 8.6 | 93.9 |
|  | Associate professor | 9 | 4.5 | 4.5 | 98.5 |
|  | Full professor | 3 | 1.5 | 1.5 | 100.0 |
|  | Total | 198 | 100.0 | 100.0 |  |

**Gender**

|  | | Frequency | Percent |
| --- | --- | --- | --- |
| Valid | women | 100 | 50.5 |
|  | men | 98 | 49.5 |
|  | Total | 198 | 100.0 |

**Age**

|  | | Frequency | Percent |
| --- | --- | --- | --- |
| Valid | 40 | 23 | 11.6 |
|  | 41 | 13 | 6.6 |
|  | 42 | 25 | 12.6 |
|  | 43 | 14 | 7.1 |
|  | 44 | 16 | 8.1 |
|  | 45 | 19 | 9.6 |
|  | 46 | 8 | 4.0 |
|  | 47 | 4 | 2.0 |
|  | 48 | 5 | 2.5 |
|  | 49 | 9 | 4.5 |
|  | 50 | 12 | 6.1 |
|  | 51 | 3 | 1.5 |
|  | 52 | 1 | .5 |
|  | 54 | 3 | 1.5 |
|  | 55 | 3 | 1.5 |
|  | 56 | 4 | 2.0 |
|  | 57 | 3 | 1.5 |
|  | 58 | 3 | 1.5 |
|  | 59 | 2 | 1.0 |
|  | 60 | 11 | 5.6 |
|  | 61 | 3 | 1.5 |
|  | 62 | 1 | .5 |
|  | 64 | 1 | .5 |
|  | 65 | 5 | 2.5 |
|  | 67 | 2 | 1.0 |
|  | 68 | 2 | 1.0 |
|  | 69 | 2 | 1.0 |
|  | 76 | 1 | .5 |
|  | Total | 198 | 100.0 |

**Years of employment at current university**

|  | | Frequency | Percent |
| --- | --- | --- | --- |
| Valid | 1 | 2 | 1.0 |
|  | 2 | 17 | 8.6 |
|  | 3 | 19 | 9.6 |
|  | 4 | 29 | 14.6 |
|  | 5 | 75 | 37.9 |
|  | 6 | 34 | 17.2 |
|  | 7 | 14 | 7.1 |
|  | 8 | 8 | 4.0 |
|  | Total | 198 | 100.0 |

**Trying to publish a paper in impact factor journal at least once**

|  | | Frequency | Percent |
| --- | --- | --- | --- |
| Valid | Yes | 92 | 46.5 |
|  | No | 106 | 53.5 |
|  | Total | 198 | 100.0 |

**Being a member of editorial board in any scientific journal**

|  | | Frequency | Percent |
| --- | --- | --- | --- |
| Valid | Yes | 70 | 35.4 |
|  | No | 128 | 64.6 |
|  | Total | 198 | 100.0 |
