# Supplementary material for: Dynamic academic networking concept and its links with English language skills and research productivity–non-Anglophone context
Source: PLoS One. 2021 Feb 2;16(2):e0245980. doi: 10.1371/journal.pone.0245980 (PMC7853504; doi:10.1371/journal.pone.0245980)
Supplement: Attachment — Submitted filename: Response to Reviewers.docx [file pone.0245980.s003.docx]

**Reply to Editor/Reviewer comments**

| **Editor/Reviewer comments** | **Reply** |
| --- | --- |
| - Text structure: Please, try to re-organise your text according to PLOS ONE's guidelines (see https://journals.plos.org/plosone/s/submission-guidelines#loc-manuscript-organization). Currently, the boundaries between the 'Materials and Methods' section with the Introduction and the Results sections are blurred. This makes the reading more difficult, as references to previous works in the literature, justification of the applied methods and presentation of the obtained results are mixed. | Thanks for suggestion.  We have revisited our manuscript and we have restructured the text into more detailed structural parts and now there are the following sections and subsections:  [Introduction 4](#_Toc40460256)  [Literature review 6](#_Toc40460257)  [Academic ties, dynamic networking and research productivity 6](#_Toc40460258)  [English language skills and research productivity 10](#_Toc40460259)  [Hypothesized research model 12](#_Toc40460260)  [Insert Figure 1 about here 12](#_Toc40460261)  [Methods 13](#_Toc40460262)  [Measures development 13](#_Toc40460263)  [Research sample 15](#_Toc40460264)  [Testing measurement quality 16](#_Toc40460265)  [Results 18](#_Toc40460266)  [Discussion 19](#_Toc40460267)  [Conclusions 24](#_Toc40460268)  [Practical implications 24](#_Toc40460269)  [Limitations and further research suggestions 25](#_Toc40460270)  [Acknowledgements 26](#_Toc40460271)  [References 27](#_Toc40460272)  [APPENDIX 32](#_Toc40460273)  Additionally, we have developed a section called: “Hypothesized research model” and associated it with figure 1 which gives a reader a quick overview into the links our research focuses on.  He hope that you like the structure better now. |
| The applied methods should be properly presented and justified. For example, what were the criteria to select the potential responders to be invited to take the survey (i.e. all the scholars above 40 years old)? | Many thanks for spotting this issue in our submission. We have followed your suggestion by incorporating changes into the way research methods are presented.  The relevant part reads as follows now:  This paper uses survey data gathered in April 2014 from senior scholars employed at universities in Poland and specializing in business-related fields, e.g. management, marketing and business logistics, human resource management, etc. Due to the absences of a relevant sampling frame, we used non-random sample of senior scholars, i.e. scholars at the age of 40 or older, because we wanted to focus only on scholars that started their primary education in the communist era, when Russian language was obligatorily taught at schools as a foreign language and it is rather a burden for them, i.e. in relation to other younger scholars in Poland exposed early to English language communication. The informants were selected mainly from biggest Polish universities with faculties related to business studies (see the list of 10 main universities in the appendix). The universities receive very good scores in ranking of universities in Poland according to a well-known ranking: ranking.perspektywy.pl. However, to increase the effectiveness of informants selection we also allowed our informants to send the survey link to their peers from other institutions and in that case we asked informants to select only most productive scholars from their social network. Thus, we can assume that final sample was skewed with regard to the normal distribution of research productivity, i.e. we selected more productive scholars than the average scholar, but that was not treated as an issue because we did not aim at representative sampling, but instead we wanted to test hypothesized model on the purposively selected sample of Polish academicians that are likely active in publishing and networking as well. We selected informants qualified as senior scholars through age filter question, i.e. only scholars that declared being in the age of 40 years old or older were selected for analysis. Some questionnaires needed to be eliminated due to being provided by younger scholars, the lack of answers to main questions and “rapid clickers” which resulted in keeping 198 questionnaires from senior scholars for further analyses. |
| How were the questions of the survey designed (e.g. why productivity is measured by blind-reviewed journals)? In relation to this, notice PLOS' publication criterion #3 (https://journals.plos.org/plosone/s/criteria-for-publication#loc-3). | We have revised the relevant section as follows:  This study follows [22], who suggest bibliometric techniques as irrelevant for investigating scholars from less developed countries, because such scholars are not well represented in international scientific databases. Additionally, using bibliometric sources such as Scopus or Googlescholar was found as irrelevant, because such databases do not provide the way to measure scholars’ networking activities except co-authorship patterns. The rationale for this study was to go to “the black box” of academic networking. Similarly, we were not able to use any publicly available sources for measuring scholars’ language skills as available proxies, e.g. publishing in English was found as too general. We also wanted to control for the influence of some additional variables, e.g. conscientiousness or perceived academic culture at the university, so we have used self-report for all research variables similarly to [22].  The research productivity was measured in the way aligned with specific features of transforming academic systems in CEE [12]. Specifically, CEE scholars are very underrepresented in top-tier journals and, on the other hand, they publish massively in non-impact factor journals and, to large extent, in Polish-medium journals and Polish-medium research monographs [36, 27]. We also followed [22] who suggested that in the context of less developed countries papers published in local and foreign journals both have some value from the perspective of academic career. To purify measurement from lowest quality publications we have clearly asked informants to indicate number of publications in “blind-review journals” with regard to last two years. Limiting perspective to output from last 2 years was done to control for the cumulative publication effect related to informants’ age differences. However, we have not limited measure of research productivity to publication output only, but we have also asked the informants about the number of external research grants they successfully received so far. In Polish academic system, similarly to many post-communist academic systems, applying for external funds for research projects is very competitive and at the same time succeeding in such competition is treated as important criterion for academic promotion and annual bonuses at scholars’ home faculties. Additionally, receiving external financing is many times “the ticket” to international visibility of a scholar, because it assures resources appropriate for visiting prestigious international conferences, financing professional language proofreading, etc. Therefore, this research uses formative measurement of research productivity as composed of two indicators: scholars’ effectiveness with regard to applying for external research grants and scholars’ effectiveness with regard to publishing papers in blind review academic journals in the last two years.  The survey measure of academic networking was inspired by research [38] which proposed item pool for networking capability in the context of inter-firm cooperation. We adapted these items to the context of academic networking on interpersonal level. The measures of the existing professional ties, namely Home network and Corporate network, were adapted from [39]: we distinguished between different types of academic partners and we incorporated self-reported measures of network quality complementing measures of network size. This method of measuring existing academic network was previously used by another research conducted in all 4 Visegrad countries illustrating that in case of all of these countries connections built by scholars are much more intensive within the boundaries of their home faculties than outside their universities [12]. Finally, English language skills were measured reflectively using one item referring to English language speaking and the other item referring to English language writing.  To measure the control variables we generally used single items or multi-items adapted from prior studies in the same area. Specifically, we adapted items provided by [24] and then replicated by [35] to measure teaching load and resource support. We used one of items proposed in previous researches [40, 41] to measure informants’ character, specifically informants’ conscientiousness as a personality variable. All other control variables; Productivity Culture, Employment time, Academic rank and Age, were generally measured as simple self-reporting scales. All items that we have used in our survey are presented in the Appendix. |
| In your Methods section, please provide additional information about the participant recruitment method and the demographic details of your participants. Please ensure you have provided sufficient details to replicate the analyses such as: a) the recruitment date range (month and year),  b) a description of how participants were recruited, and c) descriptions of where participants were recruited and where the research took place, including the names of the universities where the participates were surveyed. | Following your suggestion the methods section has been extended (see above).  Additionally, we have provided many additional tables in the appendix that present the characteristics of research participants, including:   1. Main universities 2. Contract type 3. Position at home faculty 4. Gender 5. Age 6. Years of employment at current university 7. Trying to publish a paper in impact factor journal at least once 8. Being a member of editorial board in any scientific journal |
| Please also include additional information regarding the survey or questionnaire used in the study and ensure that you have provided sufficient details that others could replicate the analyses. For instance, if you developed a questionnaire as part of this study and it is not under a copyright more restrictive than CC-BY, please include a copy, in both the original language and English, as Supporting Information. | We have decided to enlarge the description of measurement indicators by providing the table entitled “Survey questions”. This part presents all questions that we have used in the data analysis presented in the manuscript. |
| We note that you have indicated that data from this study are available upon request. PLOS only allows data to be available upon request if there are legal or ethical restrictions on sharing data publicly. For more information on unacceptable data access restrictions, please see http://journals.plos.org/plosone/s/data-availability#loc-unacceptable-data-access-restrictions. | Following the journal’s requirements, we have uploaded the Xcel file with all survey data we have used for the analysis presented in the manuscript. The database contains additional information from the online survey that allows for comparing the research results in case of potential replication, i.e. answering start date, answering finish_date & answering_time. All other scholars interested in the field would be able to download the file and replicate our PLS-SEM analytical procedure. |
| We note you have included a table to which you do not refer in the text of your manuscript. Please ensure that you refer to Table 1 and 2 in your text; if accepted, production will need this reference to link the reader to the Table. | Many thanks for spotting this. In this version we have referred in the manuscript to both: table 1 and table 2. |

**Temat:** PLOS ONE Decision: Revision required [PONE-D-20-10186] - [EMID:e211dc3ef4bdd159]

PONE-D-20-10186
**Networking and English language to sustain career in academic community under transition
PLOS ONE**

Dear Dr. Wieczorek,

Thank you for submitting your manuscript to PLOS ONE. After careful consideration, we feel that it has merit but does not fully meet PLOS ONE’s publication criteria as it currently stands. Therefore, we invite you to submit a revised version of the manuscript that addresses the points raised during the review process.

Before sending our manuscript out for review, I would like to ask you to revise some formal aspects of the text:

- Text structure: Please, try to re-organise your text according to PLOS ONE's guidelines (see https://journals.plos.org/plosone/s/submission-guidelines#loc-manuscript-organization). Currently, the boundaries between the 'Materials and Methods' section with the Introduction and the Results sections are blurred. This makes the reading more difficult, as references to previous works in the literature, justification of the applied methods and presentation of the obtained results are mixed.

- Methodology justification: The applied methods should be properly presented and justified. For example, what were the criteria to select the potential responders to be invited to take the survey (i.e. all the scholars above 40 years old)? How were the questions of the survey designed (e.g. why productivity is measured by blind-reviewed journals)? In relation to this, notice PLOS' publication criterion #3 (https://journals.plos.org/plosone/s/criteria-for-publication#loc-3).

We would appreciate receiving your revised manuscript by Jun 14 2020 11:59PM. When you are ready to submit your revision, log on to https://www.editorialmanager.com/pone/ and select the 'Submissions Needing Revision' folder to locate your manuscript file.

If you would like to make changes to your financial disclosure, please include your updated statement in your cover letter.

To enhance the reproducibility of your results, we recommend that if applicable you deposit your laboratory protocols in protocols.io, where a protocol can be assigned its own identifier (DOI) such that it can be cited independently in the future. For instructions see: <http://journals.plos.org/plosone/s/submission-guidelines#loc-laboratory-protocols>

Please include the following items when submitting your revised manuscript:

- A rebuttal letter that responds to each point raised by the academic editor and reviewer(s). This letter should be uploaded as separate file and labeled 'Response to Reviewers'.
- A marked-up copy of your manuscript that highlights changes made to the original version. This file should be uploaded as separate file and labeled 'Revised Manuscript with Track Changes'.
- An unmarked version of your revised paper without tracked changes. This file should be uploaded as separate file and labeled 'Manuscript'.

Please note while forming your response, if your article is accepted, you may have the opportunity to make the peer review history publicly available. The record will include editor decision letters (with reviews) and your responses to reviewer comments. If eligible, we will contact you to opt in or out.

We look forward to receiving your revised manuscript.

Kind regards,

Sergi Lozano
Academic Editor
PLOS ONE

Journal Requirements:

When submitting your revision, we need you to address these additional requirements.

1. Please ensure that your manuscript meets PLOS ONE's style requirements, including those for file naming. The PLOS ONE style templates can be found at

https://journals.plos.org/plosone/s/file?id=wjVg/PLOSOne_formatting_sample_main_body.pdf and

https://journals.plos.org/plosone/s/file?id=ba62/PLOSOne_formatting_sample_title_authors_affiliations.pdf

2. In your Methods section, please provide additional information about the participant recruitment method and the demographic details of your participants. Please ensure you have provided sufficient details to replicate the analyses such as: a) the recruitment date range (month and year),  b) a description of how participants were recruited, and c) descriptions of where participants were recruited and where the research took place, including the names of the universities where the participates were surveyed.

3. Please also include additional information regarding the survey or questionnaire used in the study and ensure that you have provided sufficient details that others could replicate the analyses. For instance, if you developed a questionnaire as part of this study and it is not under a copyright more restrictive than CC-BY, please include a copy, in both the original language and English, as Supporting Information.

4. We note that you have indicated that data from this study are available upon request. PLOS only allows data to be available upon request if there are legal or ethical restrictions on sharing data publicly. For more information on unacceptable data access restrictions, please see http://journals.plos.org/plosone/s/data-availability#loc-unacceptable-data-access-restrictions.

In your revised cover letter, please address the following prompts:

a) If there are ethical or legal restrictions on sharing a de-identified data set, please explain them in detail (e.g., data contain potentially sensitive information, data are owned by a third-party organization, etc.) and who has imposed them (e.g., an ethics committee). Please also provide contact information for a data access committee, ethics committee, or other institutional body to which data requests may be sent.

b) If there are no restrictions, please upload the minimal anonymized data set necessary to replicate your study findings as either Supporting Information files or to a stable, public repository and provide us with the relevant URLs, DOIs, or accession numbers. For a list of acceptable repositories, please see http://journals.plos.org/plosone/s/data-availability#loc-recommended-repositories.

We will update your Data Availability statement on your behalf to reflect the information you provide.

5. We note you have included a table to which you do not refer in the text of your manuscript. Please ensure that you refer to Table 1 and 2 in your text; if accepted, production will need this reference to link the reader to the Table.

[Note: HTML markup is below. Please do not edit.]

Reviewers' comments:


[NOTE: **If reviewer comments** were submitted as an attachment file, they will be attached to this email and accessible via the submission site. Please log into your account, locate the manuscript record, and check for the action link "View Attachments". If this link does not appear, there are no attachment files to be viewed.]


While revising your submission, please upload your figure files to the Preflight Analysis and Conversion Engine (PACE) digital diagnostic tool, <https://pacev2.apexcovantage.com/>. PACE helps ensure that figures meet PLOS requirements. To use PACE, you must first register as a user. Registration is free. Then, login and navigate to the UPLOAD tab, where you will find detailed instructions on how to use the tool. If you encounter any issues or have any questions when using PACE, please email us at [figures@plos.org](mailto:figures@plos.org). Please note that Supporting Information files do not need this step.
