# Supplementary material for: Dynamic academic networking concept and its links with English language skills and research productivity–non-Anglophone context
Source: PLoS One. 2021 Feb 2;16(2):e0245980. doi: 10.1371/journal.pone.0245980 (PMC7853504; doi:10.1371/journal.pone.0245980)
Supplement: Attachment — Submitted filename: Response to reviewers10.08.20.docx [file pone.0245980.s004.docx]

**Dear Editor and Reviewers,**

Thank you very much for providing us with constructive feedback for the last version of our manuscript. Reading carefully your comments and thinking about them many times, we understood that our manuscript was underdeveloped in many places and although we kept the main “message” and “data” of our manuscript, we have introduced many changes to the manuscript. The current version we uploaded with this submission is thus largely rewritten. We hope that you like this version better now. Below we present the way we have addressed your comments. Please take into consideration that changes we have made in writing were so substantial that it would be strange to copy-paste all elements we changed into this R2R document. Thus, our R2R describes rather in brief the changes we made, however, following PLOS One instructions, we have uploaded the manuscript in two versions: one with track changes and one without track changes, so we hope you can easily connect R2R file with all detailed changes we have introduced to the manuscript.

**Editor comments:**

As you can see below, both reviewers found the topic of your work interesting and relevant. However, they also raised a number of serious concerns. In particular, your revision should especially address the following issues:

- **Work positioning in the state-of-the-art**: Both reviewers asked to develop further the *literature review*. As stated by Reviewer 1, this is needed to precisely identify the gap the work aims to address and, eventually, frame your conclusions (see his/her comments 1) and 5) ).

Thank you. Indeed, we have found that our literature review was not sufficient and did not allow us to state our contribution clearly. We have used more than 30 more publications to improve positioning of our study in the current version. What is more important than the number of publications, is that we have especially used the recent extensive review studies, i.e. Bozeman, et al. 2013; Hall, et al. 2018; Leahey 2016, to find to what extent current Science of Team Science (SciTS) has already addressed the aspect of academic collaboration we have focused on. It allowed us to appropriately identify the novelty of our study which is discussed in many places in the manuscript including introduction, literature review and discussion. Here is how introduction positions our work now as an example:

*One of the widely acknowledged mechanisms of increasing research productivity is research collaboration, the phenomenon that has many facets and its definition has gone through some evolution in the last two decades [6,7,8]. The importance of this mechanism was discussed in so many studies that the new interdisciplinary research area was proclaimed, namely the Science of Team Science or SciTS [9]. Although SciTS provides well documented insights into how research teams are built and what factors help in effective team management, much less is known about tools and tactics that individuals may use while networking, specifically to mitigate collaboration risks and dealing with academic ties instability[6, 10]. There is also a call to integrate within SciTS various theories, and methods from across disciplines, because research collaboration is a clearly interdisciplinary research phenomenon [9]. This study corresponds with the gaps identified in the literature by proposing a “dynamic academic networking” concept (DN) that is conceptually distinct to the traditional, narrow concepts of research collaboration, but refers to wide spectrum of academic interactions which may be all perceived as important academic tasks [11, 12]. Specifically, following self-interest theories of organizational networks [13] and dynamic capabilities view in strategy research [14], we propose DN as routinized patterns of scholar behaviour that are oriented at learning and productivity development through academic relationships at various stages of their development. We also use our knowledge on the networking processes in non-Anglophone academic systems to hypothesise English language skills as an antecedent of DN in such context. Our study addressed the call for cross-theoretical and cross-disciplinary approach to understand better links between research collaboration and research productivity by using insights not only from SciTS, but also from organizational and strategy research and linguistic studies. Last but not least, our research model with DN as a focal construct is validated through two data collections, one using self-reported survey measures and the other combining some of these survey measures with Scopus based data on scholars’ productivity. Both of these studies are limited to the data gathered about scholars specialized in business science employed in Poland as a representative of non-Anglophone countries.*

- **Methodological issues**: Both reviewers raised methodological concerns (see comments 3 - 5 by Reviewer 1, and comment 3 by reviewer 2). Notice that PLOS publication criterion #3: Experiments, statistics, and other analyses are performed to a high technical standard and are described in sufficient detail." (<https://journals.plos.org/plosone/s/criteria-for-publication#loc-3>).

Thank you. We have taken the comments related to methodological aspects very seriously. To address methodological concerns, we have referred to all detailed methodological concerns raised by reviewers (see our replies below, in the part related to reviewers comments). We have largely revised the “the research method” section, “research results” section and “research limitation” section too. Most importantly, we have extended our data collection to include ex-post validity check of our first analysis that is fully based on self-reported scholars’ indications. We call it study 2 and it refers to data collected from Scopus database with regard to the fraction of the original sample that was available in Scopus due to the relatively low coverage of Polish business scholars in most known international databases. This another data collection provided strong support for our research model. Obviously, we are still aware that our research results and not without limitation and we give it full consideration in relevant final section of our manuscript, however, we believe that our study is valuable in terms of offering DN as a novel concept for which we provide initial nomomological validiy test using the data from non-Anglophone context and further research may further validate if this concept is valid with regard to other contexts and using larger datasets.

**Reviewer 1 comments**

Reviewer #1: The article concerns an important topic. Important for theoretical but also practical reasons. It is also valuable that it concerns the functioning of science outside the leading countries in terms of quantity and quality of scientific production. However, one can have numerous, serious reservations about the article in its present shape. Here are some key issues:

Thank you so much for acknowledging the importance of our research.

1) The authors emphasize that “prior studies have generally investigated academic collaboration as a “black box” and “Prior research treated academic collaboration usually a ‘black box’ focusing mainly on co-authorship while academic networking is a complex”. This statement is entirely false.

It is enough to mention the recent literature reviews about "team science", in which numerous examples of unpacking the "black box" are cited:
• Hall, K. L., Vogel, A. L., Huang, G. C., Serrano, K. J., Rice, E. L., Tsakraklides, S. P., & Fiore, S. M. (2018). The science of team science: A review of the empirical evidence and research gaps on collaboration in science. American Psychologist, 73(4), 532.
• Leahey, E. (2016). From sole investigator to team scientist: Trends in the practice and study of research collaboration. Annual review of sociology, 42, 81-100.
• Sonnenwald DH (2007) Scientific collaboration. Ann. Rev. Info. Sci. Tech. 41 (1): 643–681.
Or classical papers, as Katz JS, Martin BR (1997) What is research collaboration. Research Policy 26 (1): 1–18.

What is more, scientific cooperation studies using randomized trials have recently started to appear. For example:
• Boudreau, K., Ganguli Prokopovych, I., Gaule, P., Guinan, E. C., & Lakhani, K. R. (2012). Colocation and scientific collaboration: evidence from a field experiment. Harvard Business School working paper series# 13-023.

In this case, it is not just about the pair of missing footnotes. The problem is more serious. Without a reference to the current state of knowledge and ongoing discussions in the field, it is difficult to assess what contribution the reviewed article would make to the development of the discipline. This aspect should be carefully thought out by the authors and clearly explained in the text.

Thank you very much for spotting the underdevelopment of our writing in the previous version of the manuscript. We agree that our literature review was limited indeed as we have not even included the recent extensive SciTS review studies, i.e. Bozeman, et al. 2013; Hall, et al. 2018; Leahey 2016, to find to what extent current Science of Team Science (SciTS) has already addressed the aspect of academic collaboration we have focused on. In the current version, where our writing is largely changed in comparison to the original version, we have used more than 30 more publications to improve positioning of our study within the literature.

Yes, we agree that it is false to say that “prior studies have generally investigated academic collaboration as a “black box””, what we were arguing in the previous version. Therefore, what you may see through “track changes” in our current manuscript we have substantially improved our literature review and following other authors that also reviewed SciTS literature, we have clearly stated the research gaps that are visible in the literature. Actually, the literature review we have conducted provided us with better rationale for describing the novelty of our research which now is described mainly in terms of conceptualizing and providing non-Anglophone context validity test for the dynamic networking (DN) as a concept that addresses some gaps in the literature. We have extensively described DN concept and its novelty in many parts of our manuscript, so copy-pasting all of these parts here does not make any sense. However, again you may easily find these parts in many places including introduction, literature review and discussion section. Here we just copy few parts of the manuscript that briefly exemplify our writing:

(from introduction)

*One of the widely acknowledged mechanisms of increasing research productivity is research collaboration, the phenomenon that has many facets and its definition has gone through some evolution in the last two decades [6,7,8]. The importance of this mechanism was discussed in so many studies that the new interdisciplinary research area was proclaimed, namely the Science of Team Science or SciTS [9]. Although SciTS provides well documented insights into how research teams are built and what factors help in effective team management, much less is known about tools and tactics that individuals may use while networking, specifically to mitigate collaboration risks and dealing with academic ties instability[6, 10]. There is also a call to integrate within SciTS various theories, and methods from across disciplines, because research collaboration is a clearly interdisciplinary research phenomenon [9]. This study corresponds with the gaps identified in the literature by proposing a “dynamic academic networking” concept (DN) that is conceptually distinct to the traditional, narrow concepts of research collaboration, but refers to wide spectrum of academic interactions which may be all perceived as important academic tasks [11, 12]. Specifically, following self-interest theories of organizational networks [13] and dynamic capabilities view in strategy research [14], we propose DN as routinized patterns of scholar behaviour that are oriented at learning and productivity development through academic relationships at various stages of their development. We also use our knowledge on the networking processes in non-Anglophone academic systems to hypothesise English language skills as an antecedent of DN in such context. Our study addressed the call for cross-theoretical and cross-disciplinary approach to understand better links between research collaboration and research productivity by using insights not only from SciTS, but also from organizational and strategy research and linguistic studies. Last but not least, our research model with DN as a focal construct is validated through two data collections, one using self-reported survey measures and the other combining some of these survey measures with Scopus based data on scholars’ productivity. Both of these studies are limited to the data gathered about scholars specialized in business science employed in Poland as a representative of non-Anglophone countries.*

(…)

(from literature review)

*The SciTS has progressed substantially in the last 2 decades which is visible through the recent attempts to map its development [6, 9, 10]. Particularly, our knowledge is much advanced with regard to how scholars select partners [e.g. 24, 25] what team composition works e.g. 26, what team processes help in effective team functioning [e.g. 27, 28] and how institutional resource availability impacts on initiating and managing research collaboration [e.g. 29, 19]. However, SciTS is fragmented with relation to research disciplines and many things seem to be unsolved. First of all, the SciTS is dominated by measuring academic collaboration through co-authorship which depreciates all forms of academic collaboration that did not finish in joint publications [6, 9]. Such approach puts aside all meaningful interactions between scholars such as exchanging ideas, sharing research techniques and research data that may, but not necessarily must, result in publishing together[30]. While sharply delimiting the boundaries of what is academic collaboration and what it is not might be problematic, especially in terms of when it starts and finishes [7], the academic collaboration might be perceived broadly as all interactions between at least two scholars that help in sharing of meaning and executing academic tasks [8]. Recently, [6]defined academic collaboration in even wider terms as ‘‘social processes whereby human beings pool their human capital for the objective of producing knowledge’’ (p. 3). Such perspective gives appropriate flexibility for studying team science in their various forms and connecting these forms with research effectiveness at both levels: team effectiveness, e.g. publishing together and individual effectiveness, e.g. publishing individually using insights from collaboration. In fact, in comparison to teamwork in typical commercial organizations [31], academic collaboration at all its stages, including deciding whether to initiate it at all, is much more based on decisions of individuals being “able to act as free agents” ([9] p. 542). Identifying trends and gaps in SciTS, [10] suggests that one of the most promising avenues is looking at the collaboration from the perspective of individual scholars involved and impacting that various forms of collaboration made on them. Therefore, SciTS may benefit not only by utilizing insights from intra-organizational teamwork management literature [32, 33], but it may also use inspirations from inter-organizational relationship management literature, where business entities are perceived as being less or more capable in collaborating and applying collaboration strategies to retrieve relational rents [34, 35, 36]. Similarly scholars may apply various tactics towards their professional relationships and leverage their performance this way.*

*Secondly, SciTS is recently found as presenting too rosy picture of academic collaboration, while there is a need to prepare researchers for facing collaboration costs effectively[10]. In fact, research collaboration brings many problems which if not properly managed, may outweigh the cooperation benefits. As academic collaboration often happens between actors very different in terms of their work experience and their resources, there is a real threat of exploitation and free riding of a stronger against the weaker one, while the weaker does the vast majority of work [37]. In turn, this threat is especially important with regard to social sciences [38]. This is not a trivial problem, because the diversity as the main premise of effective team science somehow implies the power asymmetry between cooperating scholars and the equal contribution and credit related to team science cannot be ever delivered in real life and, perhaps should be not even facilitated on an institutional level. The mentoring is identified as one of typical styles of collaboration usually associated with senior scholars cooperating with junior scholars and students [39] and this style can happen always when a more experienced scholar interacts with a less experienced one. For example, such cooperation style is typical between scholars employed at the same academic positions but having very different publication records. It is also typical for cooperation between scientist from countries at very different levels of their development [40]. In general, brokering which refers to some scholars occupying the most resourceful and beneficial position in academic networks is rather a typical phenomenon [41, 42], however from the perspective of individual scholars that are not “stars” yet, there is a need to apply some ways to mitigate the risk of being exploited, or in more general terms, “to mitigate the costs of collaboration” ([10]p. 88). Logically, the scholar should manage collaboration inputs with collaboration outputs appropriately, especially in the longer time frame. They should also aim at gradually strengthening position towards being less dependent, e.g. managing their own research teams. Even without conscious attempts of one partner towards exploiting the other partner, the team science often becomes problematic due to unclear governance of research projects; the epistemic and ethical responsibility becomes too blurred and nobody feels fully responsible for the teamwork [43]. Thus, using the knowledge on business relationship cycles [44, 45, 46], all academic collaborations may be treated as instable occurrences, i.e. accepting that some of them never mature enough and some of them already lost their attractiveness and should be not maintained any more. Such approach towards collaboration seems relevant in social sciences, where the tangible resources, e.g. laboratories, dedicated to given academic teams are relatively small and scholars may engage in and disengage from various subsequent collaborations that develop slowly.*

*This work assumes that one of the ways to overcome current weaknesses of SciTS is to focus on academic “networking” instead of just academic “collaboration” from the perspective of a given scholar that functions in the “publish or perish” realm. Functioning in this context enforces proving usefulness of a scholar through publication productivity, but it also motivates for strengthening one’s own assets and building career. Here we follow [12]that proposes networking as one of major tasks of the academic researcher nowadays and defines networking as a “communication pattern” which comprises exchanging ideas, results and information between scholars via various channels that may, but not necessarily has to, involve collaborative research and collaborative publishing. Academic collaboration, in turn, takes more organized forms and usually results in joint publishing [12].*

*Although the literature offers fuzzy boundaries for using the term research/academic/scientific collaboration [7, 8] that could potentially involve communication pattern we write here about, we think that making distinction between these two terms is important, because in practice the academic collaboration is mainly measured through co-authorship [9, 10], thus it is associated by SciTS scholars rather with advanced forms of research teamwork. Such twist of a team science towards more developed acts of cooperation is visible even on a conceptual level. For example, although [8] proposes wide definition of scientific collaboration that equates collaboration with just interaction between scientists, the very same definition suggests that such interaction “facilitates the sharing of meaning and completion of tasks with respect to a mutually shared, superordinate goal” (p. 645). As we have already illustrated such “mutuality” and “shared meaning” does not need to happen in academic interactions, so we prefer to use the term “networking” to tackle the variety of interactions that happen in academic relationships and networks from the perspective of a focal actor. In turn, such focal actor being resource constrained has also large degree of autonomy with regard to “who” to network with and “how” to network, especially in social sciences that we focus on. Our understanding of networking as important academic task is aligned with [21] that emphasizes flexible interactions with peers in academia as the platform for developing new ideas and [21 illustrating empirically the differences between forms of academic cooperation across disciplines and concludes that informal interactions are significant for all disciplines and should be more facilitated in policy making. Evidence was provided that such informal academic communication might be more important than formal cooperation and co-authorship [47], especially in case of developing countries.*

*Corresponding with the idea of individual scholars being relatively autonomous in their networking decisions, especially in social sciences [8, 10], we are inspired by dynamic capabilities approach in strategy research [48, 14] and self-interest theories of organizational networks [13]. Dynamic capabilities approach suggests developing some specific capabilities that enable anticipating the opportunities and threats sensed in the environment [14]. In such strategy, the dynamic capabilities take a of form either some simple radical decisions, i.e. in rapidly changing situations, or routinized processes to handle environmental dynamics effectively, i.e. in moderately dynamic environments [49, 50]. In line with this approach, we perceive academic networking as generally happening in dynamic environment, i.e. we presume that the various forms of academic interaction, including collaborations are unstable and managing existing teamwork although very important is not the only task that the focal scholar must focus on if he/she wants to leverage his/her own productivity and success. Therefore, among various theories of organizational networks, we correspond here with self-interest theories that explain actors’ behaviour in organizational networks through their orientation at exploiting opportunities and cost minimizing rather than mutuality or group identities [13]. Consequently, we propose the concept of Dynamic Networking (DN) and we define it as routinized focal scholar patterns of interaction with other scholars that happen at various stages of collaboration cycle and are oriented at learning and leveraging productivity of a focal scholar. We understand DN not in binary terms, i.e. either one scholar having a DN or not, but instead we understand DN as a continuous feature of every scholar, with analogy to the concept of individual personality trait [51]. Similarly as there are not just agreeable or not agreeable people but all people are characterized by some level of agreeableness, scholars cannot be easily divided into the cohort of dynamic networkers and stable networkers. For example, one person might be quite active in socializing at conferences and successful in initiating some new academic contacts and that feature would suggest advanced DN, however the same person might have problems in critically evaluating existing research collaborations and that feature, in turn, would suggest limited DN. Low inclination towards downsizing in some existing relationships and moving attention towards new promising ones may come from various reasons including scholar personality, investments in current relationships, the lack of other opportunities, etc.*

2)
The research procedure, especially sampling, seems to make it impossible to draw conclusions that the authors want to draw. Anyway, the authors admit it themselves, but they draw an optimistic conclusion that in total this approach does not disqualify the study:
“Thus, we can assume that the final sample was skewed with regard to the normal distribution of research productivity, i.e. we selected more productive scholars than the average scholar, but that was not treated as an issue because we did not aim at representative sampling, but instead we wanted to test hypothesized model on the purposively selected sample of Polish academicians that are likely active in publishing and networking as well.”
I must admit that I do not understand this argument. It seems that since we deliberately choose the most productive researchers, it will be difficult to assess where productivity comes from. A similar caveat can be found in relation to the networking variable - since respondents were asked to send a survey to friends, it can be expected that it went proportionally more often to more networked people.

Many thanks for this comment. We think that we were not precise in the way we were describing our sampling and this could be, at least to some extent, the reason why it looks strange. The initial data collection happened several years ago that is why in the current version we have carefully looked again through the research notes and procedures we were able to retain from this period and we have revised the description of our sampling procedure as follows (*please consider that in the current writing we use the term study 1 and study 2, because we have managed additional ex-post data collection that we call study 2- see more details in the manuscript*):

*Study 1 uses survey data gathered in April 2014 from senior scholars employed at universities in Poland and specializing in business-related fields, e.g. management, marketing and business logistics, human resource management, etc. Due to the absence of a relevant sampling frame, we used non-random sample of senior scholars, i.e. scholars at the age of 40 or older, because we wanted to focus only on scholars who started their primary education in the communist era, when Russian language was obligatorily taught at schools as a foreign language and it is rather a burden for them, i.e. in relation to other younger scholars in Poland exposed early to English language communication. We assured that the sample is diversified in terms of rank these universities had in relevant domestic ranking of business education and the university type, i.e. either business-focused university or general university type. Specifically, we included all publicly owned universities specialized solely in business, i.e. “akademie ekonomiczne” (higher schools of economics) that received highest ranks in terms of business education in Poland. Kozminski University was included as the biggest and most prestigious private university offering business education. We also included universities of general type that focus on business education only among teaching other disciplines and we assured that here we have covered universities of the highest rank, i.e. University of Warsaw as well as universities with relatively low rank with regard to business education, e.g. University of Szczecin, University of Zielona Góra, University of Warmia and Mazury, Częstochowa University of Technology and Silesian University of Technology. To increase the effectiveness of informants’ selection we also allowed our informants to send the survey link to their peers from other institutions and in that case we asked informants to select only the most productive scholars from their social network. The surveyed universities’ structure presented in the appendix suggests that around 1/3 of all informants participating in the survey were employed at universities of a relatively low rank in business education according to a well-known domestic ranking: ranking.perspektywy.pl. The publication results declared by informants position the surveyed population as a quite typical population of business scholars in Poland - concluded from comparing publication scores of survey informants with publication scores revealed in another larger scale study of business scholars in Poland [67].*

*We selected informants qualified as senior scholars through age filter question, i.e. only scholars that declared being 40 years old or older were selected for the analysis. Some questionnaires needed to be eliminated due to being provided by younger scholars, the lack of answers to main questions and “rapid clickers” which resulted in keeping 198 questionnaires from senior scholars for further analyses. Although people (i.e. survey informants) were involved in the research, the approval of any institutional review board (i.e. relevant ethics committee) before the study began was found as not necessary due to the fact that the survey was anonymous and the research did not assume testing any form of physical or psychological treatment with regard to people involved in research. The research project was reviewed and approved by the National Science Centre in Poland which became the financing institution within the contract no. UMO-2012/05/E/HS4/02216.*

*In study 2 that was conducted in 2020 we have re-used survey data gathered in 2014 and combined the data with additional data about publication scores of the investigated informants that was acquired through Scopus. In line with the prior research suggesting that bibliometric sources are problematic for less developed countries, because such scholars are not well represented in international scientific databases [47], we have managed to get access to much smaller sample size than in study 1. Specifically, we retrieved relevant Scopus data for only 73 scholars participating in survey 1. Although, this sample is only a fraction from the initial database, it serves well as ex-post cross-validation test. Additionally, including Scopus data for the publication score in the period 1 Jan 2018 and 17 July 2020 extends the evidence used to test the hypothesized model from a cross-sectional study into a longitudinal study. Logically, routinized dynamic networking practices measured in the survey in 2014 should have long-term effects on scholars’ publication scores, especially in social science, where academic collaborations take usually much longer from their beginning until the publication results [8].*

3)
I understand reluctance regarding bibliometric indicators:
“This study follows [22], who suggest bibliometric techniques as irrelevant for investigating scholars from less developed countries, because such scholars are not well represented in international scientific databases.”
However, ultimately the authors use the bibliometric variable. The only difference is that it comes from a survey. It may, therefore, be imprecise. In the case of a sample of about 200 people, one could be tempted to manually check the list of publications (e.g. based on CVs, websites of the institutions, etc.), and the national publication database (as far as I know there is one in Poland, so you do not have to limit yourself to international sources, which in the case of non-English-speaking countries give a distorted picture of scientific production).
Adding an objective indicator of publication output would also allow controlling for co-authorship and the number of co-authors. It is essential because it can be expected that the way of counting the number of publications can significantly influence the results of the analysis (whole counting vs fractional counting).

Thank you. Following your concerns and constructive ideas we have done as much as we could to cross-validate our survey-based data with additional data collection, especially with regard to productivity measure. Please, take into consideration that our initial rationale remains the same, i.e. Polish scholars like scholars from many other non-Anglophone contexts, are largely underrepresented in most prestigious international databases, so this additional data collection that we were able to do, i.e. using Scopus database, covered only a fraction of the original sample (n=73, compared to original n=198), however this fraction data (called study 2 in our writing) allowed us to provide additional ex-post validity check of our hypotheses, so the nomological validity of dynamic networking concept was additionally illustrated. To keep the comparability of the initial dataset (n=198) with the “new dataset” (n=74) we have revised the measure of productivity as a dependent variable, which is now focused on publications in peer reviewed journals only (in recent years, not in total). This revision has downsized one of the variable links that we have initially hypothesized, i.e. the direct link between English language skills and scholar’s productivity. That is why we have rebuilt our hypothesized model into model with DN as a mediator between English language skills and productivity in non-Anglophone context. We argue that it does not change the main message of our study as it was and still is about the dynamic networking concept and its specific meaning in non-Anglophone context, thus the links with English language skills are important to position the constructs in relevant nomological network. We did not use any Polish national publication database to validate our findings, because such database contains so many different forms of publications, including even ones not appreciated by Polish academic system, as scholars themselves may upload some very low rank publications into this database. In the domestic university accreditation system so far, the external auditors have been used to classify these publications according to Ministry guide eventually. However, since introduction of University law 2.0 (“Konstytucja dla nauki) in 2018 (few years already after our initial data collection), the whole Polish academic system turns now into assessing research productivity based on publications registered in Scopus ,where these publications with established high SNIP score are more appreciated. That is also why we have used Scopus database to validate productivity measured in our additional data collection conducted in 2020. Our review of investigated scholars’ data in Scopus has revealed that indeed there is dynamic progress visible in Scopus coverage with regard to Polish business scholars however, still many scholars, especially senior scholars that we focus on in our study, are not covered there at all.

Last but not least, we have addressed your comment about “whole counting vs fractional counting” in the limitation and further research section as follows:

*Our study is also restricted in terms of using one specific measure of academic productivity, i.e. “normal count” or the number of publications authored by the given scholar [10], instead of using “fractional count”, i.e. number of publications divided by the number of authors[10], whereas the latter was found as a valuable measure of collaboration output, because few studies reported no link between collaboration/co-authorship and such productivity defined this way [101, 103]. Although future research should validate if DN as a focal construct introduced here is truly impactful with regard to other measures of productivity, we do not think that not-using fractional count disqualifies our research results. First of all, “the normal count” still seems to be the most popular productivity measure in SciTS[9]. Secondly, there are actually much more available measures than just “fractional” vs. “normal” publication count as academic productivity can be also measured through such factors as high-impact articles and citations [101, 104], research grants applications, novelty attributed to patents [9] and scholars’ career advancement [6]. It seems impossible to include all of these productivity measures in one research project, so such limitation is an attribute of every study in SciTS. Recently, [6] concluded in their extensive review of SciTS that there is a need for “more careful measurement of impacts as opposed to outputs” (p. 1). We argue that our main contribution to SciTS is in conceptualizing DN as a distinct collaboration-related research concept and providing valid and reliable survey-based measurement for this concept.*

4)
It seems to me that the methodological approach is not sufficiently justified. It is not clear why the authors immediately switch to a rather complex SEM-based approach. Wouldn't a more straightforward approach using maximum likelihood estimation for count variables (e.g. Poisson regression, negative binomial, etc.) be enough?
At a more detailed level, two potential problems can be identified. First, why the gender variable is not included in the main model but is only used in the additional analysis?
Secondly, it is worth considering the possible correlation of responses of individual people working in the same institutions. Did the authors include these possible correlations in the model? (For example, creating a multi-level model or adding dummy variables for individual institutions). Based on the reviewed text, it seems not. As a result, it is difficult to assess whether the values of the coefficients and the reported standard errors are reliable.

Thank you for your comment. Following your rationale, we have included gender variable as a control dummy variable into our main model instead of using that in ex-post gender related moderation analysis. Our results suggest that gender is neither significant predictor for research productivity, nor for dynamic networking (see in table 3).

We have decided to keep using PLS-SEM as our analytical technique and we provide the following rationale for that in the relevant section:

*To estimate the hypothesized links between variables we used partial least squares path modeling, which is also known as PLS-SEM and it is an alternative to covariance-based SEM (CB-SEM) that experienced exponential growth in terms of its applications in highly ranked journals in business research since the release of free available SmartPLS 2 software in 2005 [91]. PLS-SEM has recently become used in other areas of social science such as psychology [92, 93] and outside social sciences, in biology [e.g.94] and medicine e.g. [95]. We decided to use PL-SEM technique instead other techniques, e.g. regression, CB-SEM, due to few reasons. First of all, our research model contains mediation effect, i.e. DN is mediator between English skills and research productivity, and in general such effects cannot be modelled using traditional regression techniques. Secondly, as we used survey data in our analysis (only survey data in study 1 and mix of survey data and Scopus data in survey 2) the distribution of our data was to large extent non-normal which suggested and PLS-SEM allows for using this kind of data, while other techniques do not allow for that [96]. Thirdly, in this research we introduce Dynamic networking as a new concept, our approach is to large extent exploratory or it is an extension to the existing theories related to organizational networks and dynamic capabilities, and in such instances PL-SEM is preferred over more conservative approaches such as CB-SEM [96]. Fourthly, as our sample, especially in study 2 is relatively small (n=73) the use of PL-SEM is recommended and it “is a good approximation of CB-SEM results” (Hair, et al. 2011, p. 144). Last but not least, DN as well as several other antecedents in our model were conceptualized as variable – “reality” that cannot be directly observed but it is inferred from other variables that are observed. Specifically, DN is proposed here as a reflective latent variable which assumes that its measured indicators (5 Likert scales) are a sample of all possible indicators of DN latent constructs, i.e. dropping one indicator would usually not change the meaning of the construct [97]. Such approach to measurement is possible in PLS-SEM, while other techniques assume usually formative measurement, i.e. each indicator represents a dimension of meaning of the latent variable and dropping one dimension/indicator changes the meaning of the latent variable [97].*

5)
The conclusions, and especially the recommendations given by the authors, seem quite clichéd, it is difficult to see the value of novelty in them. We know that cooperation, in its various dimensions, translates into the productivity of scientists. We know that knowledge of English helps in publishing. We know that we should support the development of cooperation. I'd instead like to see narrow conclusions in this section, but with something new.

Many thanks for pointing at this disadvantage of our previous writing. Following extensive literature review and majorly revised positioning of our study vis-à-vis literature, we have now substantially changed the discussion section. We are not copying this part here as this part was almost fully rewritten, but we hope you will like it more now while reading in the body test of our manuscript.

6)
Finally, a small note. I have doubts about the correctness of the use of the term “lingua franca” by the authors. Dictionary definition says that “lingua franca: a language that is adopted as a common language between speakers whose native languages are different”. While the authors write:
“In the last two decades Polish academic system has gradually become more “open” (e.g. increasing participation in international conferences and top-tier journals), however, Polish language has remained lingua franca for Polish scholars (e.g. the tradition of PhD thesis writing in Polish language)”.
Bearing in mind the definition quoted, it seems that the native language spoken mostly in one country cannot be lingua franca for its native speakers.

Yes, we totally agree and the current manuscript is free from using this problematic term.

**Reviewer 2 comments**

Reviewer #2: An interesting paper that sheds light on the networking patterns in a non-English academic community. The text is well written and clear in terms of argumentation line and hypotheses testing. I suggest accepting this paper with some minor clarifications as follows.

Thank you so much for appreciation of our work. Following your comments and the comments of Reviewer 1, in this submission we provide a revised version of the manuscript. Although, many parts were rewritten to address all reviewers’ comments, we would like to emphasize that we did not change the main “message” of our writing and we still use the same data, only enriching it with additional ex-post validity-check data collection.

1. If possible, please provide some correspondence between your sample and available statistics on human resources in science and technology in Poland as it may help to understand which particular groups you represent in your study.

Many thanks for your comment. We have provided some revision into the manuscript following your suggestion. We have restricted the text to outline a separate section, where we describe the research context of our study to better present the academic group we represent in our study. This part is entitled now *Post-communist business science in Poland as a non-Anglophone research context*.

We have also provided there some general information about what this academic group looks like in terms of its scientific productivity, including recent changes, for example:

*It should be also mentioned that similarly to some other post-communist countries, Polish academic system, including business science or social sciences in large terms, experienced dynamic changes in the last decade. It is connected with institutional changes that have for example increased the importance of impact factor publications in the university domestic accreditation system. Although the scholars from humanities and social sciences were traditionally less published, i.e. in terms of publication numbers and, especially, less represented in international databases as compared to representatives of Polish scientist from other disciplines, e.g. medicine or informatics, analysing publication activities of Polish scholars from social sciences and humanities in recent years, [80] found that the percentage of non-publishing scholars has decreased enormously and when it comes to number of publications Polish scholars in this disciplines become similarly productive as scholars from more advanced West European countries. Among all analysed disciplines related to social sciences, the scholars specialized in business were found as most productive. Nevertheless, this shift towards increased productivity is visible only in terms of publication quantity, whereas publication quality in terms of impact factor publications is still relatively low and it appears to be best in psychology among all analysed social sciences.*

2. It would be great to see some references to similar or related studies from other posts-soviet countries you are mentioning in your analysis if you know any.

As our study focuses on dynamic networking as a new construct and we explain in the manuscript why this concept provides the new angle to SciTS literature, there were no prior studies that have tested or even discussed conceptually the importance of this construct in the context of post-soviet countries. Nevertheless, we have made references to several studies that explain specific collaboration-related behaviour and research productivity in non-Anglophone countries context, including:

Ynalvez, M. A. and W. M. Shrum (2011). "Professional networks, scientific collaboration, and publication productivity in resource-constrained research institutions in a developing country." Research policy 40(2): 204-216

Wieczorek AL, Mitręga M. Academic teachers under stress in the publish or perish era. 1st ed. Warsaw: CeDeWu; 2017

Mitręga, M., Ed. (2016). Networking akademicki jako czynnik produktywności pracowników naukowych Uniwersytet Ekonomiczny w Katowicach.

Mitręga M, Spáčil V, Hetesi E, Prónay S, Wieczorek A L, Hvizdová E, et al. Leveraging the success of young Visegrad scholars in business discipline. 1st ed. Warsaw: CeDeWu; 2014

Curry, M. J. and T. M. Lillis (2010). "Academic research networks: Accessing resources for English-medium publishing." English for Specific Purposes 29(4): 281-295.

Curry, M. J. and T. Lillis (2013). A scholar's guide to getting published in English: Critical choices and practical strategies, Multilingual matters.

Kulczycki, E. (2019). "Wzory publikacyjne polskich naukowców w latach 2013–2016: nauki humanistyczne i nauki społeczne."

3. I still have some doubts about the use of bootstrapping to increase your sample size from 198 to 5000 cases. Do you have any ways to assess trace fraction or bootstrapping was used only for probability distributions, and you don't try to represent any particular social group from the population?

Thank you so much

In our bootstrapping PLS-SEM analysis we did not aim at extracting any specific sub-sample like, in case for instance of inter-group moderation testing, but instead we just followed the standard recommendations with regard to model estimation which suggest testing results in both versions which is 500 bootstrap samples and 5000 bootstrap sample treating the later one as more “accurate” or “stable” path significance result. Here we cite exemplary recommendations from relevant literature:

- “*Use bootstrapping to assess the path coefficients’ significance. The minimum number of bootstrap samples is 5,000*” (Hair, et al. 2011 p. 145)
- *The default is 500 leave-one-out samples, with replacement. This default is suitable for exploratory purposes. For confirmatory purposes, a larger number (e.g., 5000) is recommended, though this will take more computational time*. (Garson 2016 p. 93)

Garson, G David

2016	Partial least squares: Regression and structural equation models. Asheboro, NC: Statistical Associates Publishers.

Hair, J. F., Christian M Ringle, and Marko Sarstedt

2011	PLS-SEM: Indeed a silver bullet. The Journal of Marketing Theory and Practice 19(2):139-152.
