# Supplementary material for: Dynamic academic networking concept and its links with English language skills and research productivity–non-Anglophone context
Source: PLoS One. 2021 Feb 2;16(2):e0245980. doi: 10.1371/journal.pone.0245980 (PMC7853504; doi:10.1371/journal.pone.0245980)
Supplement: Attachment — Submitted filename: Reply to reviewers_23.12.2020.docx [file pone.0245980.s005.docx]

PONE-D-20-10186R2
Dynamic Academic Networking Concept and its Links with English Language Skills and Research Productivity - non-Anglophone Context
PLOS ONE

Reviewer #2

Thank you for addressing my early comments and revising your manuscript in line with the recommendations of both reviewers. I read the text carefully and found your paper much more consistent. However, due to rewriting a few points appeared I want to draw your attention.

At page 1 you say: «Logically, in comparison to other disciplines team social science is also very risky because social science research methods are less standardized and the researchers’ cultural backgrounds are more meaningful due to interpretative epistemology bringing more potential for team conflicts». Thought this argument seems reasonable it doesn’t come from the observation that «collaborative research projects in social science are much longer [8].» Please either provide some evidence for this or reconsider your statement in a more neutral manner.

First, thank you very much for appreciating our revision.

Thank you also for spotting a blurred logical sequence in two sentences in the introduction. It was improved in the current version.

You pay attention to the importance of English language in collaboration, but do not specify whether this collaboration should be international (should it be?). I am also not sure that your example of science industry collaboration (see page 17) is relevant to the topic of your research as it might take place with local producers.

Many thanks for your comments.

The main causal path (these that we focus on) are these presented in figure 1 (copied below). Only in the control variables we present and test the other connections, e.g. Corporate network -> Productivity; Home network -> Productivity. As we are introducing the new concept of “dynamic networking” to establish its nomological validity and also to assure that this concept is really distinct in relation to other concepts established in literature, we have included some other network-based control variables but we do not concentrate on them in our research.

We are fully aware that the science - industry collaboration that we discuss in the paper (again with regard to control variables and to distinguish our focal construct from other constructs) may take local form only and we do not suggest in the manuscript that we focus on international science - industry collaboration. In the specific discipline and institutional context that our research is focused on, such international science - industry collaboration is extremely rare. We have focused on the population of scholars at the age of 40 or older in post-communist countries so concerning their habits and competences built largely in the communist time, they may have some ties with business practitioners but on local scale only and these ties were built mainly in the 90s., i.e. at the beginning of transformation period with fastest growing markets and these ties were used mainly as a secondary, very effective source of extra money for them, e.g. through commercial research projects.

Similarly, the collaboration that we mainly focus on as represented by our focal construct of Dynamic Networking (see figure 1) is not about international networking only but it is rather about some activities that systematically expand work connections of focal scholars beyond their home faculty network (e.g. *“I try systematically to broaden the area of my research contacts through initiating contacts with new people at national and international conferences”*) and it is also about pragmatic approach towards existing network (e.g. “*I systematically evaluate the usability of my current research contacts (e.g. taking into consideration such benefits as joint publications*”). In the current version of the manuscript we give full consideration to these issues, including providing the definition of dynamic networking as *routinized focal scholars’ patterns of dynamic interaction with other scholars that happen at various stages of collaboration cycle and are oriented at learning from others and purposively shaping network of a focal scholar*.

Therefore, in our study we do not focus on “international networking’ but rather “dynamic networking” that goes beyond home network and includes, but is not limited to, international networks. We also make a distinction between network/collaboration on one hand and networking on the other in several parts in the manuscript. However, we still argue that English language is an important antecedent to dynamic networking even beyond networking on international scale, because language capabilities are the passage to good quality, effective team science nowadays, and specifically for the senior scholars in non-anglophone context.

For example, the relevant part reads as follows:

Many inhabitants of non-Anglophone countries not only have worse command of English, but they also perceive the world differently which is caused by general cultural differences and also by the language they speak and by absence of some notions in their native language which, in turn, may make some phenomena difficult to understand for those people. Sapir–Whorf hypothesis [59, 60] explains differences in human cognition [61] and emotion [62] through differences in human language. In the context of academic interactions, it means that the way the scholar constructs his/her worldview is based on the language the scholar acquired and uses. Extrapolating this mechanism to the concept of DN, it could be that acquiring English language skills makes non-Anglophone scholars not only more capable of English-language based communication with other scholars, but also it may change their approach to interaction with other scholars. Specifically, as English language is perceived a language rich in pragmatics [63], the acquisition of English language may make a non-Anglophone scholar more pragmatic in interaction with other scholars, which enable DN and indirectly, it has positive effect on scholar’s productivity. Furthermore, taking into consideration that we build our understanding of the world through the lens of our native language, the lack of some words in a given language influences the way people think and act as its native users . We may take the notion of *networking* as an example of it. In Polish language there is no equivalent of the English word *networking* and many people simply do not know the word/concept at all. The notion is known by some business people, scientists or researchers who study this phenomenon, but they speak and write about it borrowing the English term, so one can assume they do not have a preunderstanding of the concept – those who do not do research on networking may even not know what networking really entails and as a result do not have proper abilities to network with others. In turn, a term *collaboration* which is closely connected with *networking*, but of course is not synonymous, has negative connotations for some Poles as it in their minds this notion may refer mainly to the traitors of the nation collaborating with Germans at the times of II World War (such WWII practices were called *collaboration, “kolaboracja”* in Polish). This can make some scholars associate the concept of collaboration with unethical cooperation practices (like e.g. ghostwriting) and limits their understanding of the wide spectrum of networking actions that are available, i.e. initiating new connections with scholars for mutual learning.

The section on English skills and dynamic networking can be shortened and more focused on the arguments why English matters (other languages may also be important) instead of providing a list of non Anglophone countries :)

Thank you for this comment, we believe that following the course of action you suggested will make the section more logical. Indeed, we tried to shorten the text wherever possible – for instance we deleted the list of non-Anglophone countries and tried to once again explain why English language is considered a global language now (in general and as a global language of science). It was not, however, possible to shorten the further parts of the section (i.e. the part where we explain the context of post-communist countries and the impact of communist times on linguistic skills of senior scholars) as we felt that we should explain it in detail why people from such countries may be disadvantaged linguistically due to post-communist heritage of their countries of origin and living. We also felt it was necessary not to shorten the part referring to Sapir-Whorf hypothesis as it explains how a language we speak influences the way we perceive the world and how we think generally and we present there how it could have impacted the attitudes of post-communist scholars towards e.g. networking.

At page 23 you mention scholars that may have «have strongest networks and easiest access to resources» and call them «brokers». I find the term «gatekeeper» would be more suitable for such a definition as it refers to the network control function rather than to the service provision. I might haven’t got your point here.

Ok, thank you. We have changed terms here.

I don’t have questions about Study 1, but for Study 2 I support the other reviewer’s comment on the necessity of data verification& I suggest looking not only at Scopus which is English language bias but also at other open sources that can provide additional information about publication activity of Polish authors, e.g. ResearchGate, Academia.edu. Even Google Scholar can be relevant here as long as you try to assess the overall progress of particular authors and make a longitude of it. Otherwise provide stronger arguments why Scopus remains the only relevant source for your study.

Thank you. We understand your concern. Actually it is difficult to find the balance between or even combine the Polish post-communist academic system with typical Western Anglophone academic systems, where top-tier journal publications are almost only in centre of attention when it comes to research productivity measurement. However, we would like to emphasize that Polish academic system, including Polish business science where our study is located, underwent dynamic changes in the last decade, which made it much closer to Western systems of productivity measurement. Our study is based on data gathered at different points of time for the dependent variable: 2014 (study 1) and 2020 (study 2). For study 1, the wide focus on productivity measured through publications of various kind is understandable, while the focus on only Scopus registered publications in the study 2 was also a very conscious choice considering the institutional changes observed in last decade. To explain our rational we have extended relevant part of the manuscript and this part reads as follows now:

The pace of changes of Polish academic system, similarly to other post-communist systems of CEE (Central and Eastern Europe) has substantially progressed in the last decade. Analysing the publication patterns of non-English-speaking European countries: Czech Republic, Denmark, Finland, Flanders (Belgium), Norway, Poland, Slovakia, and Slovenia, [81] found that in the period between 2011 and 2014 the publication patterns were stable in West European and Nordic countries, whereas CEE countries experienced considerable changes towards more and more emphasis in English language publications in international journals. For Poland as the biggest CEE country the trend continues with regard to impact factor publications which is largely based on the strong impact of policy instruments with regard to country’s national research evaluation system, academic promotion procedures and competitive grants [82]. In October 2018 the new Law on Higher Education and Science was established in Poland (also called *Science Constitution 2.0* to emphasize the depth of institutional changes) which centralized leadership of the university authorities [83] and at the same time imposed much more demanding measures of scientific productivity based on publication outputs in Scopus database. Specifically, the new ranking of publications in journals was mostly based on SNIP (The Source-Normalized Impact per Paper) indicator which is a relatively new journal metric by Elsevier's Scopus [84]. The SNIP was selected because this indicator acknowledges citation potential related to differences in topicality across scientific fields. Therefore, it may be concluded that in contrast to previous periods, nowadays the research productivity of business scholars in Poland is evaluated mostly through their publications registered in Scopus. This institutional change motivated Polish scholars to publish more and more in highly ranked journals, so it may be assumed that the trend identified for the period 2011-2016 [81, 82] received some additional impetus. It translates into even more challenging situation for the older generation of Polish scholars who started their careers in communist period, where publications in Polish language were the main pattern of dissemination of their research results.

I still don’t understand why connections with industry should have an effect on publication activity. This line of argumentation remains unclear to me

Thank you. Again, please notice that the academic-industry ties have been introduced into the model as control variable only, so it means that the focal causal path does not include any influence from such ties on publication activity. We have actually presented the line of argumentation explaining that existing academic-industry ties do not impact on publications effects due to specific context of our research (post-communist “experienced scholars” with some business connections that are utilized mostly as source of extra income only). However, we have included this aspect as a control variable because prior research has tested this connection and found some empirical support for it. Following your concern the relevant section related to control variables was slightly revised and it reads as follows now:

In turn, Corporate network corresponds with University-industry collaboration concept [8] from the perspective of an individual scholar and refers to professional relationships between focal scholar and business practitioners. This specific cross-industry collaboration was perceived as a driver of publication productivity in prior research, because it increases the innovativeness of academic research [85, 86], however as explained before in this study, it is treated as a control variable only, due to specific the institutional context of our research (see previous section).

Reviewer #3:

The paper is clear and argumentative. The fact that worldwide research is English based is an interesting subject of research, especially when can help scholars to reflect on their career. The historical contextualization in scholars that have been affected by the presence of the Berlin Wall is relevant in a context where English is now required to spread research. It is also pleasant to see references on European policies at this regard, as international mobility is a valuable form of both cultural and linguistic integration. Employed methods shows clearly the impact of past education in Poland, which should recognize the role of EU in the last decades. In that sense, it would be interesting to employ the same methods to analyze other languages that in the future can be predominant in research environments, such as Chinese, Portuguese, and Indians, e.g. I simply say that the method can be further validated with a different point of view. Thanks for the pleasant reading.

Dear Reviewer, thank you so much for your kind attitude towards our study. We totally agree that there are many ways that our findings may be validated and expanded in further research. We have proposed several further research directions in the last section of our manuscript.

When it comes to Figure 1 which you claimed was missing, we double-checked and it was uploaded to the system. It was, however, uploaded in eps format (according to PLoS instructions) and maybe for that reason you could not see it. We copied the figure to this file (see above, p. 2).
